# The time between intention and action affects the experience of action

**DOI:** 10.3389/fnhum.2015.00366

**Published:** 2015-06-19

**Authors:** Mikkel C. Vinding, Mads Jensen, Morten Overgaard

**Affiliations:** ^1^Cognitive Neuroscience Research Unit, Center of Functionally Integrative Neuroscience and MindLab, Aarhus UniversityAarhus, Denmark; ^2^Centre for Cognitive Neuroscience, Department of Communication and Psychology, Aalborg UniversityAalborg, Denmark

**Keywords:** intention, intentional binding, motor planning, proximal intention, distal intention, delayed intentions, sense of agency, experience of action

## Abstract

We present a study investigating how the delay between the intention to act and the following action, influenced the experience of action. In experiments investigating sense of agency and experience of action, the contrast is most often between voluntary and involuntary actions. It is rarely asked whether different types of intentions influence the experience of action differently. To investigate this we distinguished between proximal intentions (i.e., intentions for immediate actions) and delayed intentions (i.e., intentions with a temporal delay between intention and action). The distinction was implemented in an intentional binding paradigm, by varying the delay between the time where participants formed the intention to act and the time at which they performed the action. The results showed that delayed intentions were followed by a stronger binding effect for the tone following the action compared to proximal intentions. The actions were reported to have occurred earlier for delayed intentions than for proximal intentions. This effect was independent of the binding effect usually found in intentional binding experiments. This suggests that two perceptual shifts occurred in the contrast between delayed intentions and proximal intentions: The first being the binding effect, the second a general shift in the perceived time of action. Neither the stronger binding effect for tone, nor the earlier reports of action, differed across delays for delayed intentions. The results imply that delayed intentions and proximal intentions have a different impact on the experience of action.

## Introduction

In the present study, we investigated how the intermediate delay between intention about a forthcoming action, and the subsequent action, influenced the experience of action. To achieve this we distinguished between proximal intentions and delayed intentions. Proximal intentions are characterized by being immediately transformed into action when they are formed (Searle, 1980). Delayed intentions are formed, maintained in memory, and then realized into action at a later point in time (Gilbert, 2011). The intentions are formed in the same manner for both types of intentions. The difference is whether the intention is realized immediately or realized at a later time.

When making a voluntary action, we experience it as different from passive or involuntary actions (Tsakiris and Haggard, 2005). Voluntary actions are accompanied by the experience that we cause and control our own actions, a so-called sense of agency (Bayne and Pacherie, 2007). How the intention to make a voluntary action influence the experience of action is not well understood. Experimental approaches that investigate experience of action often distinguish between self-initiated actions and actions that are cued or externally triggered, e.g., by transcranial magnetic stimulation (Jensen et al., 2014). Such approach does not tell us whether different types of intentions affect the experience of action in different ways. Experiments investigating self-initiated actions usually consist of participants performing spontaneous actions when they feel the first intention, or “urge,” to execute the action (following the instructions from Libet et al., 1983). This type of task must be characterized as investigating proximal intentions (Zhu, 2003; Mele, 2010). Though understanding of how proximal intention relate to action is an important aspect of voluntary action control, it is of equal importance to understand how different kinds of intentions relate to action.

Pacherie (2008) has proposed a theoretical model of how intentions are organized in a structure of hierarchical cognitive processes. The cognitive processes behind action generation and experience of action constitute the lowest level in the model. While these processes are mostly automatic they are guided by proximal intentions, which constitute a higher level in the hierarchy. Longer lasting and abstract intentions are found at the highest level in the hierarchy. Higher order intentions work as a global set of parameters that guide action and influence the action specific proximal intentions, as well as the processes of action generation and processes generating the experience of action.

If the distinction between proximal and delayed intentions follows such hierarchical organization they are to be considered two different types of intention involving different cognitive functions. However, proximal and delayed intention could be the same cognitive process only extended in time. If this is the case, then the distinction between delayed and proximal intentions are merely conceptual, and they are better thought of as the same type of intention.

A few studies have investigated the difference between proximal intentions and delayed intentions (Vinding et al., 2013, 2014)^1^. Participants were instructed to either act immediately, or wait a certain time-interval before acting, when they experienced the intention to act. To investigate if delayed intentions affected the experience of action, we used an Intentional Binding paradigm, with the addition of conditions where participants formed the intention to act, but delayed the action. Intentional Binding was first described by Haggard et al. (2002), who showed that when participants made voluntary actions that caused a tone, the tone and the action were perceived, as closer together in time compared to non-voluntary actions. This effect is now known as intentional binding, and is used as an implicit measure of agency (Moore and Obhi, 2012; Jensen et al., 2015). Intentional binding is defined as a shift in perceived time of an action toward the effect of the action, and a complementary shift in perceived time of effect of action toward the time of action (Haggard et al., 2002). The Intentional Binding paradigm is an example of a method for investigating the experience of action using proximal intentions. In our previous experiment we added a delayed intention condition, to investigate the difference between delayed and proximal intentions in the binding effect. This showed a stronger binding effect for the tone following actions for delayed intentions compared to proximal intentions (Vinding et al., 2013).

The categorical distinction between proximal and delayed intentions attempts to separate cases where an action is performed immediately after the intention is formed, or whether there is an intermediate delay. In previous experiments, a fixed intermediate delay was used between the time, intentions were formed, and the time, the intentions were realized into actions (Vinding et al., 2013, 2014). It was not addressed if the effect of delayed intentions changed as a consequence of the delay period *per se*. We therefore included a series of delays between intention and action. This specifically investigated whether the previously shown effect of delayed intentions should be taken as evidence for two different kinds of intention with different functional properties, or as evidence for a general effect of delaying responses. Thus, it was possible to test if the delay between intention formation and action influenced the experience of action.

**FIGURE 1:**
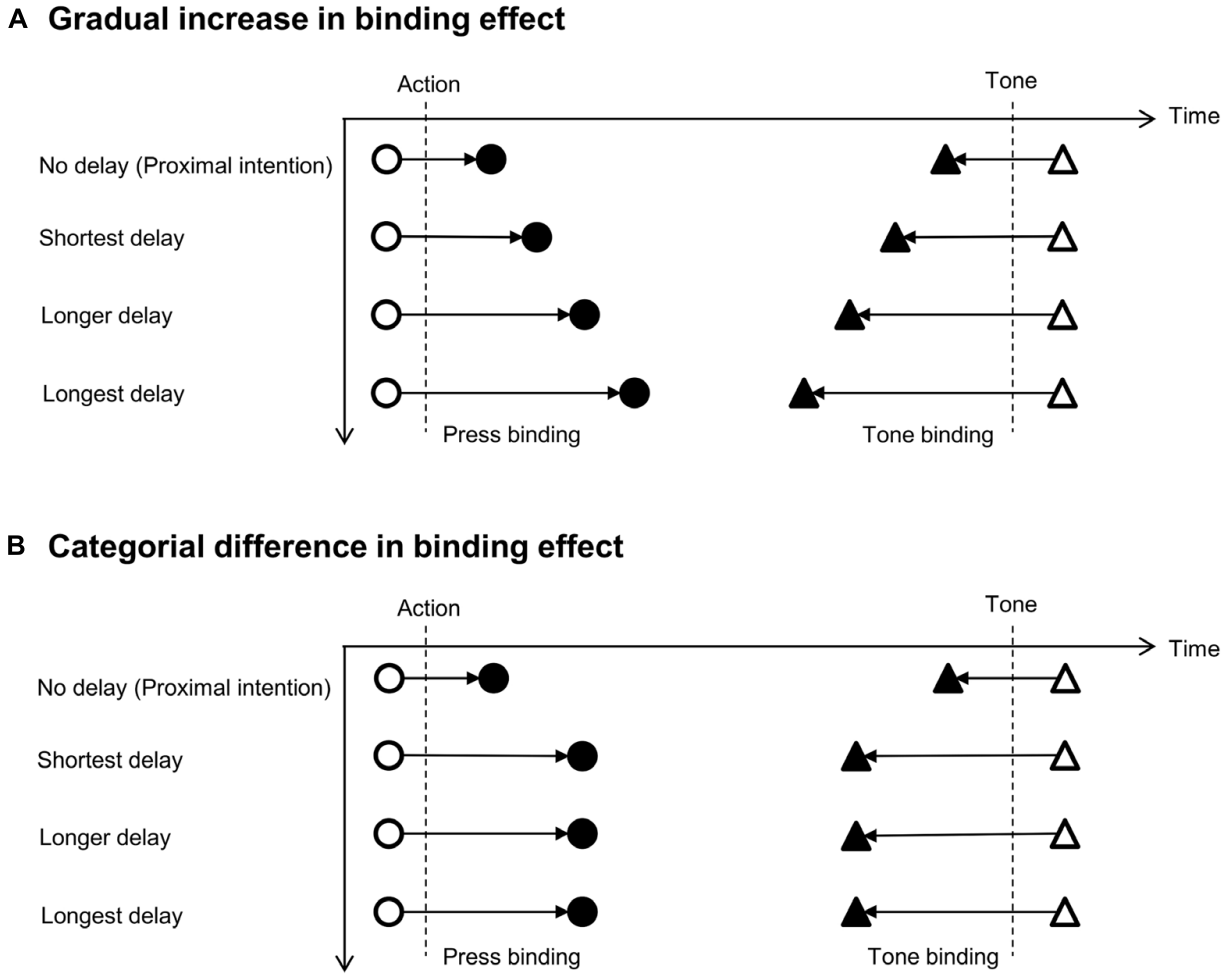
**Schematic illustration of the two hypotheses about the difference in binding effect across delays between intention and action.** The binding effect is defined as a shift from baseline in the perceived time of action and tone. Press is marked by circles and tone is marked by triangles. Open symbols indicates baseline and solid symbols indicate operant conditions. **(A)** If a difference in binding effect between delayed and proximal intentions was a result of the intermediate time between intention and action, the binding effect would be expected to increase gradually following the delay between intention and action. **(B)** If delayed and proximal intentions are different processes with different impact on the experience of action not dependent on time, we would expect a categorical difference between proximal and delayed intentions.

If a difference in binding effect between the two conditions is a general effect of delay between intention formation and action, the binding effect would be expected to increase gradually as the delay between intention and action increased. The proximal intention would show the least binding effect and the longest delay would exhibit the greatest binding effect (**Figure 1A**). However, if the difference is a result of two (at least partially) distinct cognitive processes, a categorical difference in the binding effect between delayed or proximal intended actions would be expected (**Figure 1B**). In this case, we would expect differences between proximal intentions and delayed intentions, but not between different delays for delayed intentions.

## Experiment 1

### Materials and Methods

Fifteen participants between 19 and 26 years (mean: 23.1 years, 10 female) participated in the experiment. All participants gave written consent after being informed about the procedure of the experiment. The experiment took about 2 h. Participants received 200 DKK for participating in the experiment. The Central Denmark Region Committees on Health Research Ethics provided written confirmation that no ethical approval was required according to Danish law.

**FIGURE 2:**
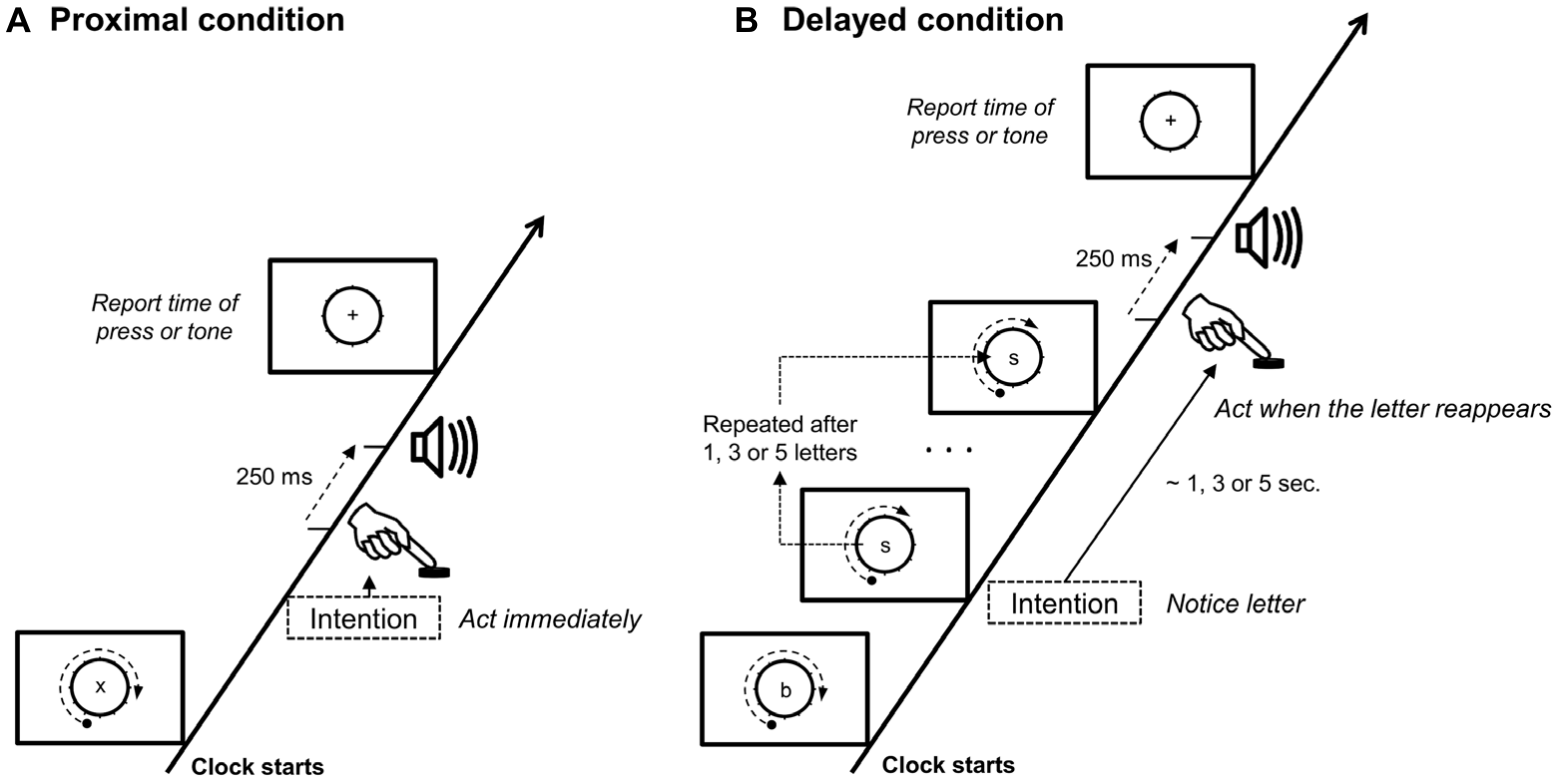
**Schematic illustration of the experimental conditions.** Each trial started when the clock began to run. Letters were shown in the middle of the clock one at the time for 800 ms, followed by 200 ms blank. **(A)** In proximal conditions participants pressed the key as soon as they had the intention to act. **(B)** In the delayed conditions participant had to notice the letter, when they had the intention to act, and then press when the same latter was presented again. In all operant conditions a tone were played 250 ms after the press. After a short random delay, participants had to report the “time” on the clock, when they either made the action or heard the tone.

The experimental set-up was based on a typical intention binding paradigm (Haggard et al., 2002). The action was the same for proximal and delayed intentions. In the *proximal conditions*, the action was executed immediately, whereas in the *delayed conditions*, the action was delayed (see **Figure 2**). Participants formed both the proximal and delayed intentions at their own pace. This matched the usual instructions given in intentional binding experiments (Haggard et al., 2002; Moore and Obhi, 2012). Retrieval of delayed intentions was cue-based.

In all trials participants watched a clock with a dot rotating on the edge at 2560 ms per rotation. The periphery of the clock had a visual angle of 2°. The action consisted of participants pressing a key on a standard computer keyboard with the right hand. When the key was pressed a single tone (1000 Hz for 100 ms) were presented 250 ms later in headphones worn by the participants. Stings of letters were presented in the center of the clock, one letter at the time. The string of letters was generated for each trial in a pseudorandom manner so all letters were repeated one, three or five letters later. Each letter was shown for 800 ms followed by 200 ms blank before the next letter was shown. Clock and letters were shown in white on a black background. The presentation of letters was pseudo-randomized, so each letter was repeated after one, three, or five letters. The paradigm was programmed and ran in PsychoPy (v. 1.79.01, Peirce, 2008).

The task consisted of two instructions about how to plan and execute the action (**Figure 2**). In the *proximal conditions* participants had to press the key immediately when they had the intention to act. Participants were specifically asked not to plan in advance when to press the key. In the *delayed conditions*, participants had to form the intention to press the key, similar to the proximal condition, but instead of executing the action immediately, they should notice the shown letter and delay the key press until the same letter reappeared. The delay between the intention and action execution would always be either one, three, or five letters long, corresponding to a delay of 1, 3, or 5 s. After each trial participants reported either the time on the clock where they made the action or where they heard the tone (see **Table 1**).

**Table 1:** Experimental conditions.

Conditions	Events	Delay	Report	Block repetitions
**Press conditions**				
Delayed – Baseline	Press	1, 3, or 5	Press	3
Delayed – Operant	Press + Tone	1, 3, or 5	Press	3
Proximal – Baseline	Press	0	Press	1
Proximal – Operant	Press + Tone	0	Press	1
**Tone conditions**				
Delayed – Operant	Press + Tone	1, 3, or 5	Tone	3
Proximal – Operant	Press + Tone	0	Tone	1
Baseline	Tone	–	Tone	1

In the delayed conditions, participants had to store one letter in memory when they formed the intention to act and keep it until they had performed the action. To minimize possible confounds, the letters were shown in all conditions. Participants were instructed to focus on the letters, even when not needed to perform the task. It was possible that the task of remembering the letter for delayed intentions interfered with the subjective reports by reducing precision of the reported times. In intentional binding the difficulty of the task can be assessed by comparing the variance in the reported times between the different tasks (Jensen et al., 2015). We therefore conducted Pitman–Morgan tests, to test for unequal variance across delays, before proceeding to the main analysis.

The proximal and delayed conditions consisted of operant and baseline conditions. In operant conditions, the tone always followed when a press was made. The baseline conditions for press reports were identical to the operant conditions except the press was not followed by the tone. The instructions were identical to the operant conditions. For tone reports there was a single baseline condition for both delayed and proximal conditions. The tone was played at a random time between 2.5 and 7 s after trial start, without participants pressing the key. Participants were instructed to wait for the tone and afterward report the time it occurred.

The different conditions were presented in blocks consisting of 40 trials each. Each delayed conditions were repeated three times (see **Table 1**), to ensure an approximately even amount of trials between the proximal conditions and the different delays in the delayed conditions. This gave a total of 13 blocks (520 trials). The order of blocks was randomized for each participant.

Before the experiment began, participants completed 10 training trials for both delayed and proximal conditions, half of which were tone reports and half of which were press reports. Participants were encouraged to take breaks between each block. Participants were instructed to mark trials in which they failed to comply with the instructions in any way, e.g., by missing target letters, by pressing an “error-key.”

For each trial the “judgment error” was calculated as difference between the reported time and the actual recorded time of the event by the computer. Due to an error, the time the tones were played was logged incorrect. Hence, only responses from the press conditions were analyzed. The tone conditions were repeated in a second experiment (Experiment 2). The repetition of the tone conditions in a new experiment was not a concern, as it has been shown that the binding effect depends on partially different processes for tone and press (Moore and Fletcher, 2012; Wolpe et al., 2013).

Trials in which participants reported they made an error, and trials where the letter shown at press was not similar to the letter shown one, three, or five letters earlier, were removed. Trials where the judgment error were greater than 400 ms from the participant’s median judgment error were classified as outliers and removed. One whole block from a single participant was excluded as the participant had removed the headphones. An average of 5.4% (range: 9–13.4%) of trials were discarded per participant due to errors. 1.2% (range: 0–4.7%) trials were discarded due to extreme values.

Data was analyzed by mixed-effect regression models using R (R Core Team) and the lme4 package (Bates et al., 2014). The regression models were fitted to the judgment error for all individual trials by full maximum-likelihood estimation. The full model contained the factor *delay* [zero delay (proximal condition), one-letter delay, three-letter delay, and five-letter delay], the factor *condition* (baseline or operant), and the interaction between the factors. Significance testing was done by removing predictors one by one from the full model, starting with the highest order interaction. Models with and without each predictor were then compared by log-likelihood ratio tests. In addition to the predictors of interest the full model included *trial order* and *block order* to control the effect of the experiment duration. Both these predictors were centered on their means so coefficients of the model reflected the average influence of these effects. To test whether the duration of the experiment or the unequal number of blocks of proximal and delayed conditions the full model included the interaction between block order, delay and condition. The random effects structure contained the factors delay and condition and individual slopes for the effect of block order for each participant.

### Results

The tests for unequal variance across delays did not suggest that any of the delayed conditions were more difficult than the proximal condition (all *p*-values in range *p* = [38.91] for the operant conditions, and all *p*-values in range [10.92] for baseline conditions). None of the participants reported to have noticed the letters were systematically presented one, three, or five letters later when directly asked after the experiment.

**FIGURE 3:**
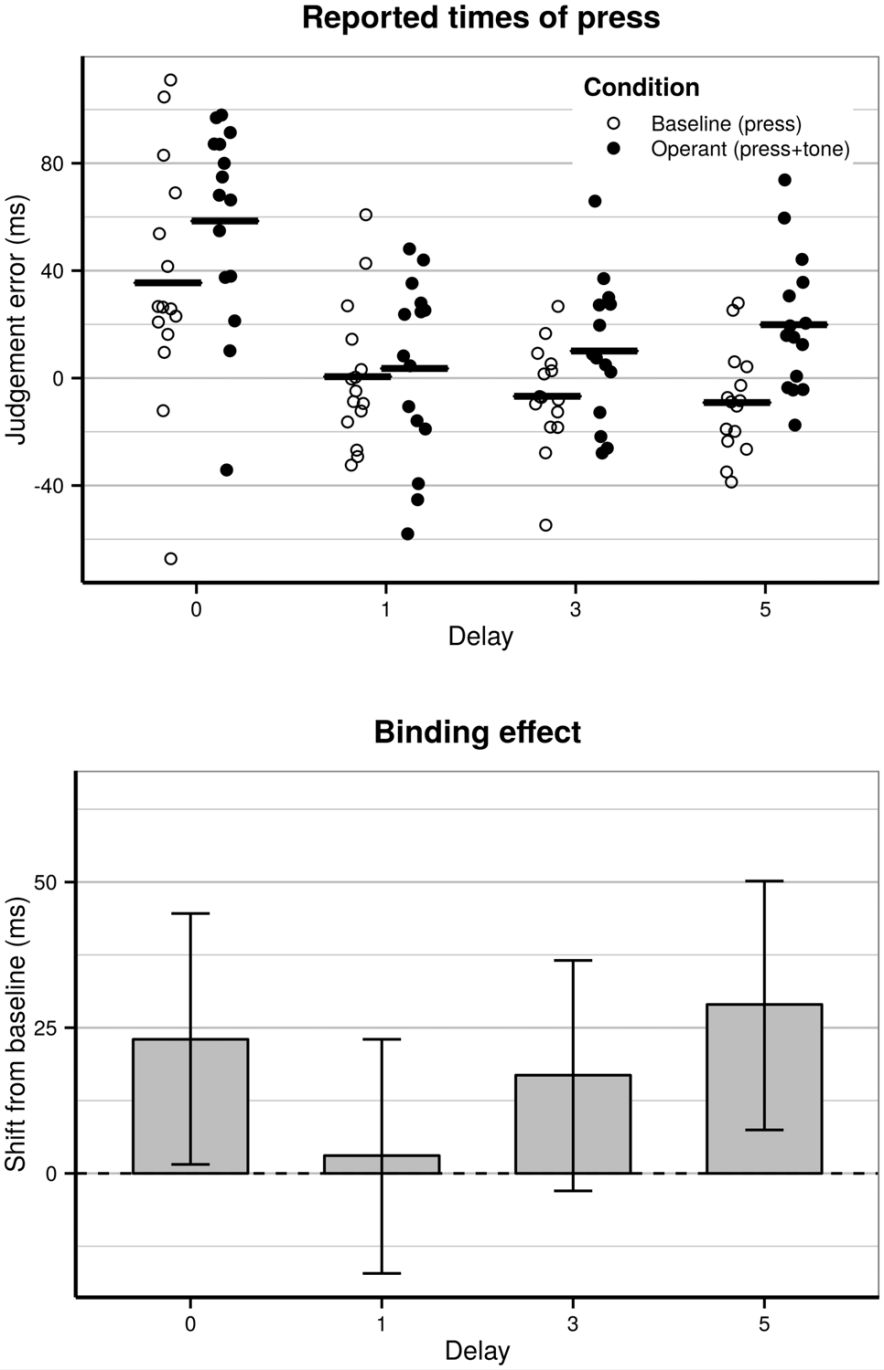
**(Top)** Reported times of action relative to the actual occurrence of action, across delays for the baseline and the operant conditions. The dots show the estimates for each participant, and the solid bars show the group level times. **(Bottom)** Group level estimated binding effect for press. Error-bars indicate 95% confidence intervals of the estimated effects based on posterior simulation of the full model (*n* = 10000).

Model comparison showed significant effect of condition [χ^2^(1) = 4.6, *p* = 0.031]. The coefficient for condition has a positive sign, which confirms that a binding effect is occurring (see **Figure 3**, bottom). There was a significant effect of delay [χ^2^(3) = 20.3, *p* = 0.0001] and the interaction between delay and condition was significant [χ^2^(3) = 10.2, *p* = 0.017]. In addition to the main effects of interest there was a significant effect of trial number [χ^2^(1) = 6.9, *p* = 0.008], and a significant effect of the interaction between trial number and block order [χ^2^(1) = 4.1, *p* = 0.044], but no significant effect of block number alone [χ^2^(1) = 0.17, *p* = 0.68]. There were no significant effects of the interactions between block order and delay [χ^2^(3) = 4.5, *p* = 0.21] or block order and condition [χ^2^(1) = 1.9, *p* = 0.17]. There was a significant effect of the three-way interaction between block order, condition and delay [χ^2^(3) = 24.6, *p* < 0.0001]. As block order and trial order were centered on their means, all other parameters reflect the “average” effect of experiment duration. The group level reported times of press estimated from the model are presented in **Table 2**. Coefficients of the full model are presented in the Supplementary Table S1.

**Table 2:** Reported occurrence of the press for operant conditions and baseline conditions, and the binding effect indicating the shift from baseline to operant condition, for all subjects.

	Operant (action + tone)	Baseline (action only)	Binding effect
Zero delay (proximal)	58.5 ms [13.3:103.9]	35.5 ms [-10.6:82.1]	23.0 ms [1.5:44.6]
One-letter delay	3.6 ms [-41.6:49.7]	0.5 ms [-44.4:47.2]	3.1 ms [-17.2:23.0]
Three-letter delay	10.1 ms [-35.6:56.1]	-6.8 ms [-52.3:39.3]	16.8 ms [-3.0:36.6]
Five-letter delay	19.9 ms [-25.4:66.3]	-9.1 ms [-55.2:37.8]	29.0 ms [7.5:50.2]

Numbers in brackets are 95% confidence intervals estimated by posterior simulation of the full model (*n* = 10000).

Goodness of fit was assessed by conditional goodness of fit, describing the overall fit of the model, and the marginal goodness of fit giving the variance explained by the fixed effect alone (Nakagawa and Schielzeth, 2013). The full model explained 44% of the total variation in the data (conditional *R*^2^ = 0.44). There was large variance between intercepts for individual subjects (SD = 79.6 ms), but less variation on individual intercepts within factors. The fixed effects alone explained 4% of the total variation (marginal *R*^2^ = 0.04).

The significant interaction between delay and condition meant that the binding effect was different between the different delays (**Figure 3**, top). Wald χ^2^ tests (De Rosario-Martinez, 2013) were performed to test what drove the interaction between delay and condition. This showed significant differences in binding effect between proximal and one-letter delay [χ^2^(1) = 4.3, *p* = 0.038] and between five-letter delay and one-letter delay [χ^2^(1) = 7.5, *p* = 0.006]. No other significant differences were found in binding effect between delays (all *p* > 0.19). The binding effect was weaker for one-letter delay compared to the proximal condition and five-letter delay.

To test what drove the significant effect of delay, tests were conducted between all delays for both baseline and operant conditions. This showed significant differences between the zero delay condition and all other delays in both baseline conditions [0–1: χ^2^(1) = 7.3, *p* = 0.007, 0–3: χ^2^(1) = 10.7, *p* = 0.001, 0–5: χ^2^(1) = 11.2, *p* = 0.0008], and operant conditions [0–1: χ^2^(1) = 18.3, *p* < 0.0001, 0–3: χ^2^(1) = 14.6, *p* = 0.0001, 0–5: χ^2^(1) = 8.9, *p* = 0.003]. No other significant differences were found between delays for neither operant nor baseline conditions (all *p* > 0.21). The time of action in the proximal condition were reported later than all delayed conditions. This did not differ between delays (**Figure 3**, top).

### Discussion of Experiment 1

The results showed differences in binding effect between delays, but only between the one-letter delay and the longest delay, and the one-letter delay and proximal conditions. The most striking finding was that the reported times of press were earlier in all delayed conditions than in the proximal conditions. The baseline time for the intentional binding was earlier in the delayed conditions compared to the proximal condition. This means that two kinds of “temporal shifts” occurred in the experience of action. The first shift was the binding effect, where the reported occurrence of action was shifted from the baseline toward the tone, as reported many times before (for review, see Moore and Obhi, 2012). The second shift was a shift in the baseline timing of action between proximal and delayed conditions.

It was hypothesized that there would be a gradual increase in binding effect as the delay between intention and action increased, if longer time was the underlying reason for the difference. If delayed and proximal intentions were different types of intention influencing the experience of action different ways, then a categorical difference were expected. The differences in baseline between proximal and all delays but not between any other delays, supports the “categorical” hypothesis. This result were, however, unexpected, as our hypotheses were about the binding effect, defined as the shift from baseline, not about the baselines alone.

The differences in the binding effect did not match either of the two hypothesized scenarios. There was no gradual increase in binding effect starting with proximal condition and increasing as the delay increased. Neither were there a clear categorical difference between the proximal condition and the delayed conditions in the binding effect. A gradual increase might be hinted starting at the one-letter delay and increasing as the delay increased, but the results are not conclusive. However, if this were the case, then it would mean that our hypothesis that delay between intention and action, only applies for delayed intentions. It does not apply for proximal intentions that are immediately realized into action.

## Experiment 2

### Materials and Methods

Twelve new participants (aged 20–26 years, mean: 23.1 years, 6 female) were recruited. All gave written consent after being informed about the procedure of the experiment. The experiment lasted for about 1 h. Participants received 100 DKK for participating.

The participants went through the tone conditions of the paradigm described above, consisting of three different instructions (see **Table 3**). In the proximal condition, participants had to press the key immediately when they had the intention to act. In the delayed conditions, participants had to form the intention to press the key and delay the key-press until the same letter reappeared. A tone (1000 Hz for 100 ms) was played 250 ms after the key-press in both proximal and delayed conditions. In the baseline conditions, the tone was played at a random time within 2.5 to 7 s after trial start, without participants pressing the key. In all conditions, participants had to report the time they heard the tone.

**Table 3:** Experimental conditions.

Tone conditions				
Conditions	Events	Delay	Report	Block repetitions
Delayed – Operant	Press + Tone	1, 3, or 5	Tone	6
Proximal – Operant	Press + Tone	0	Tone	2
Baseline	Tone	–	Tone	2

The length of the blocks was reduced from 40 trials per block to two blocks with 20 trials per block. This gave two blocks of proximal conditions, six blocks of delayed conditions, and two blocks of baseline conditions. The order of the blocks was randomized for each participant.

Participants completed five training trials for each condition to become familiar with the instructions. Participants were instructed to mark any trials where they failed to comply with the instructions by pressing an “error-key.” Participants were encouraged to take breaks between the blocks.

The judgment error, i.e., difference between the reported time and the recorded time of the tone was calculated for each trial. Trials were rejected following the same criteria as in Experiment 1. An average of 2.6% (range: 0–5.0%) of trials were discarded and 2.4% (range: 0–11.5%) trials were discarded due to extreme values.

Data was analyzed by mixed-effect regression. Models were fitted by full maximum likelihood estimation to the judgment errors using all trials. The main factor *condition* had five-levels containing zero-delay (proximal condition), one-letter delay, three-letter delay, five-letter delay, and the baseline condition. Trial order, block order, and the interaction between delays and block order, were included in the model to test for the effect of the duration of the experiment. Both trial order and block order were centered on their means. Individual intercepts were fitted for each participant for condition and individual slopes for block order. Significance testing was done by model comparisons following the same procedure as Experiment 1. The differences between proximal and the one-, three- and five-letter delays were investigated by planned *post hoc* comparison.

### Results

The Pitman–Morgan tests for unequal variance between conditions showed a significant difference between baseline and the longest delay (*t* = -2.5, *p* = 0.03). There were no significant differences between any other combinations of conditions (all *p*-values in range *p* = [11.79]). Though the longest delay was more difficult than the baseline, there was nothing that suggested that reporting the delayed conditions were more difficult than the proximal condition.

**FIGURE 4:**
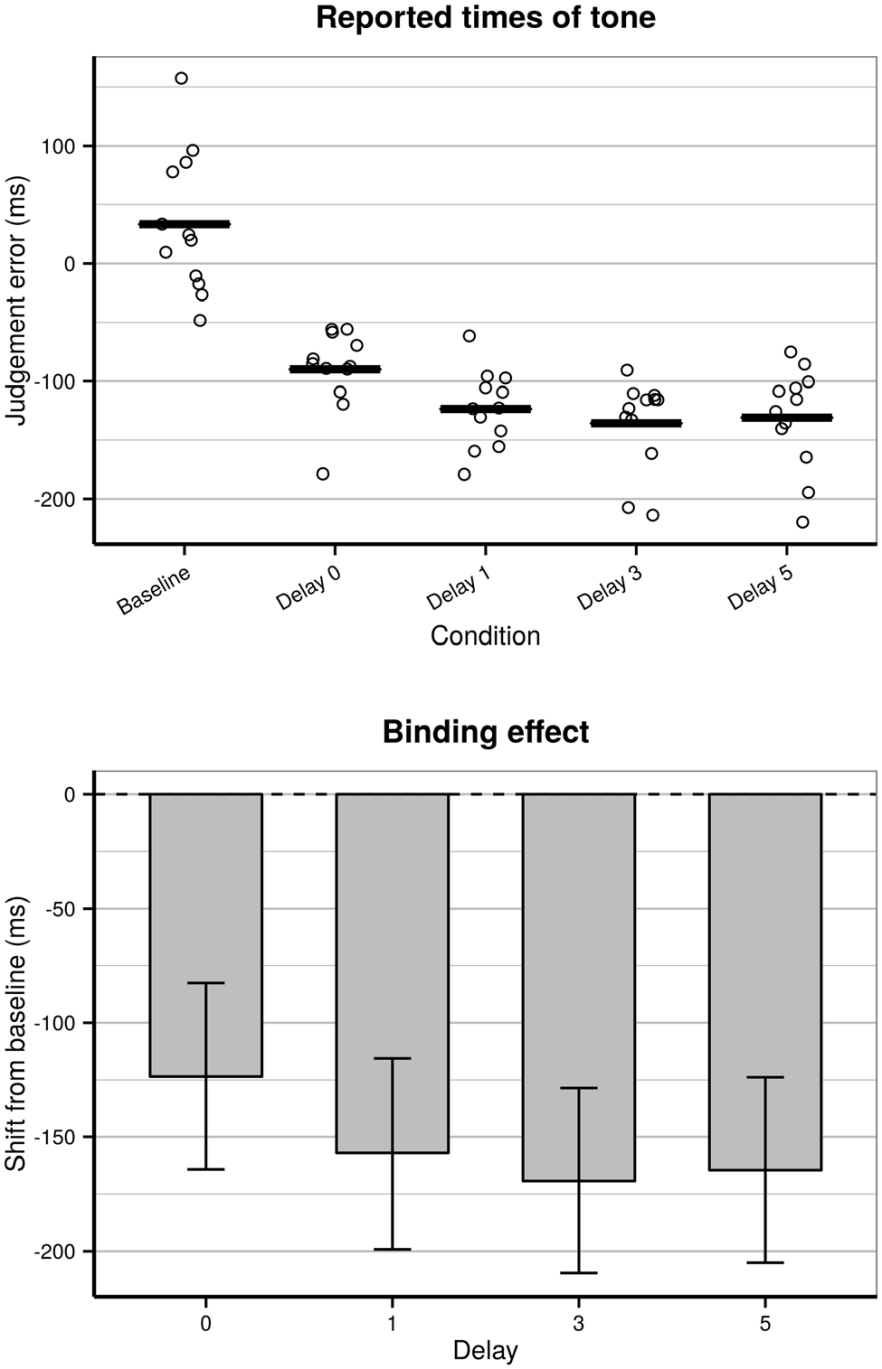
**(Top)** Reported times of the tone relative to the actual occurrence of tone, for baseline condition, proximal condition, and the different delays. The dots indicate the individual time of report for each participant and the solid bars indicate the group level estimates. **(Bottom)** Group level estimated binding effect for tone in Experiment 2. Error-bars indicate 95% confidence intervals of the estimated effects based on posterior simulation (*n* = 10000).

Model comparison showed a significant effect of condition [χ^2^(4) = 51.7, *p* < 0.0001]. The coefficients showed earlier judgment of the occurrence of tone for all operant conditions compared to the baseline condition, which confirms the binding effect in the tone conditions (see **Figure 4**). There was no significant effect of trial number [χ^2^(1) = 0.07, *p* = 0.79], probably due to the shorter block length than Experiment 1. There was significant effect of block order [χ^2^(1) = 4.3, *p* = 0.038] but no significant interaction between trial number and block order [χ^2^(1) = 0.06, *p* = 0.81]. There were no significant interaction between condition and block order [χ^2^(4) = 6.8, *p* = 0.15]. The effect of experiment duration did not differ between conditions. The group level reported times of tone estimated from the model are presented in **Table 4**. Coefficients of the full model are presented in the Supplementary Table S2.

**Table 4:** Reported occurrence of the tone for operant conditions and baseline condition, and the binding effect indicating the shift from baseline to operant condition, for all subjects.

	Operant (action + tone)	Baseline (tone only)	Binding effect
Zero delay (proximal)	90.0 ms [-150.2: -28.4]	33.5 ms [-27.7:94.0]	123.5 ms [-164.2: -82.7]
One-letter delay	-123.6 ms [-185.0: -62.3]	33.5 ms [-27.7:94.0]	157.1 ms [-199.2:115.7]
Three-letter delay	-135.8 ms [-195.7: -74.7]	33.5 ms [-27.7:94.0]	169.3 ms [-209.6: -128.5]
Five-letter delay	-131.0 ms [-191.0: -71.6]	33.5 ms [-27.7:94.0]	164.5 ms [-205.0: -123.8]

Numbers in brackets are 95% confidence intervals estimated by posterior simulation of the full model (*n* = 10000). The baseline condition was the same for all delays.

The model explained 57% (conditional *R*^2^ = 0.57) of the variance in the data. There was a large variance between individual subjects (SD = 95.4 ms) and relatively large variance in the individual intercepts within condition (SD = 47.5 ms). The fixed effects alone accounted for 15% of the variance in data (marginal *R*^2^ = 0.15).

Comparison between levels in the factor condition showed that all operant conditions were significantly different from the baseline condition (all *p* < 0.0001). The difference between zero delay and the delay of one letter was not significant [χ^2^(1) = 2.5, *p* = 0.11]. There were significant differences between zero delay and three-letter delay [χ^2^(1) = 5.0, *p* = 0.026] and between zero delay and five-letter delay [χ^2^(1) = 3.9, *p* = 0.048]. There were no significant differences across delays in the delay conditions (all *p* > 0.56). The binding effect was stronger for the three-letter and five-letter delays than in the proximal condition. The binding effect did not differ between the different delays in the delayed condition.

### Discussion of Experiment 2

The binding effect for tone did not follow the same pattern across delays as we found for press reports in Experiment 1. In Experiment 2, we found substantial binding effect in all operant conditions. The binding effects were strongest for delayed intentions. This finding is consistent with the previous study showing stronger tone binding for delayed conditions (Vinding et al., 2013).

In the present study it was further tested whether the binding effect for delayed intentions varied across different delays between intentions and actions. No differences were found in the reports of tones between the different delays within the delayed conditions, only between proximal and delayed intentions. The results did not support the hypothesis that gradually increases in delay would lead to a gradual increase in binding effect. The results fit better to the “categorical” hypothesis.

## General Discussion

**FIGURE 5:**
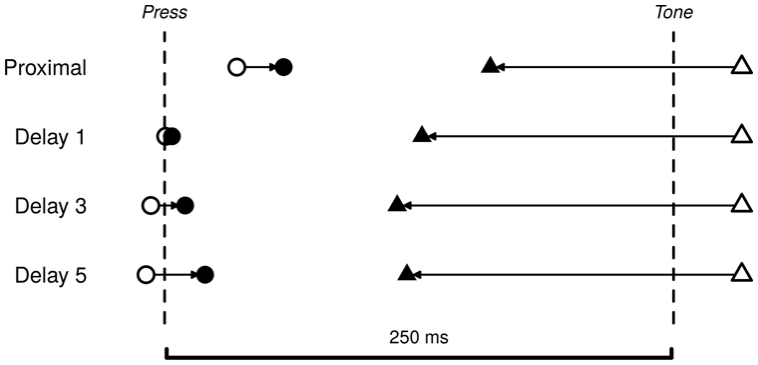
**Group level reported times of press and tone.** Press is marked by circle and tone is marked by triangles. Open symbols indicate baseline conditions and solid symbols indicate operant conditions. Arrows indicate the shift from the baselines to the operant conditions that define the binding effect. The vertical dashed lines are the actual occurrence of press and tone 250 ms apart.

The experiments showed that the temporal delay between intention and action lead to changes in the reported occurrence of actions and their effects. For neither the reports of action nor the reports of tone did we find support for the hypothesis that there would be a gradual increase in the binding effect. The results do support the hypothesis that there would be a categorical difference between delayed and proximally intended actions. This difference was most notable for the tone reports, where delayed intentions were followed by a stronger binding effect compared to proximal intentions. The binding effect did not differ between delays for delayed intentions. The binding effect for action did not clearly support any of our initial hypotheses. However, the difference in the baseline between proximal intentions and all the delayed intentions, which did not differ between delays for delayed intentions, imply a categorical difference (see **Figure 5**).

The binding effect did not follow the same pattern for tone and press. This is neither a new nor an uncommon finding. Wolpe et al. (2013) introduced an uncertainty in the tones that followed actions in an intentional binding paradigm by partially masking the tone with auditory noise. This affected the binding effect for tone but not the binding effect for press. Similar disassociations in intentional binding between press and tone have been induced by TMS to pre-supplementary motor area only affecting press binding (Moore et al., 2010), and by transcranial direct current stimulation to left parietal cortex which only affected tone binding (Khalighinejad and Haggard, 2015). The cognitive mechanism behind intentional binding and the experience of action have been proposed to be a result of integration of multiple feedback cues from different modalities that are weighted according to the certainties of the different feedback signals (Moore and Fletcher, 2012; Synofzik et al., 2013). In accordance with this view the present results suggest that delayed and proximal intentions affected the binding effect differently for the two different modalities.

The results show evidence for two perceptual shifts: The first shift was the binding effect between action and effect (i.e., intentional binding) and second shift was in the baselines between proximal and delayed conditions. The occurrence of both action and tone were reported earlier in the delayed conditions compared to the proximal conditions. This could mean that the baseline shift in the times of action for delayed intentions drove the earlier occurrence of the tone for delayed intentions in the tone conditions. The earlier reported occurrence of the tone is a joint effect of the earlier reported time of action. Both are shifted to an earlier time together as a pair. Several authors have proposed that delayed intentions involve prospective predictions or representations established before action is initiated, occurring outside the processes in immediate relation to action execution, that functions as global parameters for action selection (Pacherie, 2008; Hommel, 2009; Synofzik et al., 2013). Though longer lasting intentions often also is referring to abstract intentions beyond simple motor intentions, our results could be interpreted as a similar effect of delayed intentions for specific actions. Delayed motor intentions shifted the experience of action and tone to an earlier time.

However, due to the different baselines from which the binding effect is defined for tone and press reports it is impossible to determine, whether the earlier reported time of tone for delayed intentions should be seen as a single binding effect, or the sum of intentional binding and an additional shift following earlier perception of action. Tone reports had a single common baseline, in which participants had to await the tone without a preceding intentional action. In the press conditions, each delay had a corresponding baseline condition. For press reports, participants made an action in the baseline conditions, whereas the baseline condition for tone was passive.

Certain limitations are present in this study related to making inference on the difference between delayed and proximal intentions. How much delay there can be between a delayed intention and action is not limited to the time-scale used in the experiment. The maximum delay in our study was 5 s. The temporal delay was limited to make the experiment feasible. Subjects could have delayed intentions about doing an action after 10 s, after minutes, or days, after a year, and so on. Though the results imply a categorical difference between delayed and proximal intentions rather than a dependency upon the delay itself, how this generalizes to longer delays is uncertain.

The delayed intention task engages prospective memory in a way that the proximal intention task does not. Memory load could therefore be a possible confounding factor explaining the difference between delayed and proximal intentions. Memory load has been shown to lower judgments of agency when actions are performed while under high attentional load (Hon et al., 2013). As intentional binding is taken as an implicit measure of agency, it must be hypothesized that working memory load would decrease the binding effect. This is not what we observe. We interpreted this, as memory load was not a confounding factor when measuring the experience of action for delayed intentions. Intentional binding has previously been investigated in combination with additional visual stimuli without introducing any apparent artifacts in the results (Engbert and Wohlschläger, 2007; Desantis et al., 2011; Moretto et al., 2011).

Here we showed that for self-paced intentions, the experience of actions is dependent on the delay between intention and action. We used a cued realization of delayed intentions. Other ways of retrieval and realization of delayed intentions, e.g., time based or self-initiated retrieval (McDaniel and Einstein, 2000; West and Krompinger, 2005; Gilbert et al., 2009), might be followed by different experience of action. Whether the difference between self-paced proximal and delayed intentions also generalizes to other types of intention, such as abstract intentions, is a topic for future studies.

Given these limitations, the difference in perceived time of action and effect is evidence in favor of delayed and proximal intentions having different functional impact the experience of action. Though the relation between different types of intention and action has not been thoroughly investigated, we have argued for a difference in the experience of action by way of the simple distinction between proximal and delayed intentions. This also highlights that in order to fully understand intentions and the experience of action, future studies need to take into account that intention does not seem to be a unified concept.

## Conflict of Interest Statement

The authors declare that the research was conducted in the absence of any commercial or financial relationships that could be construed as a potential conflict of interest.

## Footnotes

1 In the cited papers, what we here refer to as “delayed intentions” was called “distal intentions.” Elsewhere distal intention also refers to abstract intentions beyond simple motor intentions (Pacherie, 2008; Mele, 2010; Synofzik et al., 2013). We therefore adopt the designation “delayed intention” for intentions about specific actions that are formed, then stored in memory, and later realized (following Gilbert, 2011).
