# Supplementary material for: The time between intention and action affects the experience of action
Source: Front Hum Neurosci. 2015 Jun 19;9:366. doi: 10.3389/fnhum.2015.00366 (PMC4473004; doi:10.3389/fnhum.2015.00366)
Supplement: Supplementary file 1 [file Table_1.PDF]

Supplementary material

Table S1: Coefficients, standard errors (S.E.), and 95% confidence intervals (CI) for the fixed effects and variance of the random effects in the regression model in Experiment 1. Numbers in the first column denote different predictors and letters denote levels within predictors. The first level in predictors is used as intercepts. Coefficients of the other levels are the change from the first level of that predictor. 95% CIs were estimated by posterior simulation of the full model (n = 10000). * indicates that CIs does not contain zero.

**Fixed effects**	**Coefficients (β)**	**S.E.**	**95%CI**
1a. Delay 0 (proximal)	35.5	29.3	[-10.6:82.1]
1b. Delay 1	-35.0	13.0	[-60.1:-9.8] *
1c. Delay 3	-42.3	12.9	[-67.2:-16.9] *
1d. Delay 5	-44.6	13.3	[-70.4:-18.2] *
2. Condition (operant)	23.0	10.9	[1.5:44.6] *
3. Trial nr.	-.4	.1	[-.6:-.1] *
4. Block nr.	-1.0	2.5	[-6.0:4.0]
5a. Delay 1 × Condition	-12.0	9.7	[-38.8:-1.2] *
5b. Delay 3 × Condition	-6.2	9.5	[-24.7:12.3]
5c. Delay 5 × Condition	6.0	9.5	[-14.0:25.8]
6. Trial nr. × Block nr.	.1	.04	[.004:.1] *
7. Condition × Block nr.	9.8	3.1	[3.9:15.9] *
8a. Delay 1 × Block nr.	-.3	2.5	[-5.1:4.6]
8b. Delay 3 × Block nr.	5.2	2.4	[.7:10.0] *
8c. Delay 5 × Block nr.	3.9	2.6	[-1.1:9.1]
9a. Delay 1 × Condition * Block nr.	-8.1	3.6	[-15.3:-1.3] *
9b. Delay 3 × Condition * Block nr.	-15.6	3.5	[-22.5:-8.7] *
9c. Delay 5 × Condition * Block nr.	-13.7	3.7	[-20.9:-6.6] *
**Random effects**	**SD**	**Variance explained**	
Delay per subject (intercept)	29.9	.05	
Condition per subject (intercept)	22.1	.03	
Block nr. per subject (slope)	6.0	.001	
Subject	79.6	.34	

Table S2: Coefficients, standard errors (SE), and 95% confidence intervals (CI) for the fixed effects and variance of the random effects in the regression model in Experiment 2. Numbers in the first column denote different predictors and letters denote levels within predictors. The model was construed so that the baseline condition was used as reference level. The coefficients for the remaining four levels are therefore directly interpretable as the group-level binding effect. 95% CIs were estimated by posterior simulation of the full model (n = 10000). * indicates that CIs not containing zero.

**Fixed effects**	**Coefficient**	**SE**	**95%CI**
1a. Baseline (single tone)	33.5	31.2	[-28.2:95.7]
1b. Delay 0 (proximal)	-123.5	20.7	[-163.7 -83.9] *
1c. Delay 1	-157.1	21.2	[-199.1:-114.7] *
1d. Delay 3	-169.2	20.6	[-209.5:-128.7] *
1.e Delay 5	-164.5	20.8	[-204.9:-123.6] *

2. Trial nr.	.1	.4	[-.7:.9]
3. Block nr.	6.0	2.6	[.8:11.1] *
4. Trial nr. × Block nr.	.03	.1	[-.2:.3]
**Random effects**	**SD**	**Variance explained**	
Delay per subject (intercept)	47.4	.1	
Block nr. per subject (slope)	8.6	.003	
Subject (intercept)	95.1	.4	
